# A Mobile App Designed to Promote Shared Decision-Making in the Treatment of Psychotic Disorders: Feasibility and Acceptability Study

**DOI:** 10.2196/68813

**Published:** 2025-07-11

**Authors:** Mari Skoge, Sofie Ragnhild Aminoff, Elizabeth Ann Barrett, Gina Engen Bryhni, Kristine Kling, Kari Jorunn Kværner, Ingrid Melle, Erlend Mork, Carmen Simonsen, Linn Nathalie Støme, Josina Vink, Tor Gunnar Værnes, Kristin Lie Romm

**Affiliations:** 1 Early Intervention in Psychosis Advisory Unit for South East Norway, Division of Mental Health and Addiction, Oslo University Hospital, Sognsvannsveien 21, Bygg 12, 2. etg, Oslo, 0372, Norway, 47 93624119; 2 Faculty of Medicine, Institute of Clinical Medicine, University of Oslo, Oslo, Norway; 3 Early Intervention in Psychosis Unit, Sanderud, Division of Psychiatric Health Services, Innlandet Hospital Trust, Ottestad, Norway; 4 Department of Strategy and Entrepreneurship, BI Norwegian Business School, Oslo, Norway; 5 Institute of Design, Oslo School of Architecture and Design, Oslo, Norway

**Keywords:** shared decision-making, patient involvement, psychotic disorders, mobile apps, mHealth

## Abstract

**Background:**

Strengthening shared decision-making in mental health care may improve the quality of services and treatment outcomes, but its implementation in services for severe mental disorders is currently lacking.

**Objective:**

This study aims to explore the feasibility and acceptability of iTandem (University of Oslo), a mobile app designed to promote shared decision-making in the treatment of psychotic disorders. In addition, the study aims to investigate mechanisms that potentially contribute to the intended effect of the app. iTandem is a therapy supplement that facilitates patient involvement in decisions regarding treatment goals and focus areas. It is designed for personalized use and contains 8 optional modules: sleep, medication, recovery, mood, psychosis, activity, substance use, and feedback concerning therapy.

**Methods:**

Patients undergoing assessment or treatment for psychotic disorders and their clinicians were recruited for the study. Patients and clinicians jointly used iTandem as part of standard treatment in a 6-week trial. We used a mixed-methods study design with a clear emphasis on qualitative methods. Feasibility and acceptability were assessed through descriptive statistics based on preintervention and postintervention questionnaires and app usage data, in addition to text responses to open-ended items. We conducted a reflexive thematic analysis of postintervention interviews to elaborate these measures and to explore mechanisms potentially contributing to achieving shared decision-making when using iTandem.

**Results:**

A total of 9 patients and 8 clinicians completed the trial. The participants evaluated iTandem as a user-friendly and acceptable tool, but there were considerable variations in how the app was integrated into treatment and in perceptions of its clinical value. The thematic analysis suggests that iTandem has the potential to facilitate shared decision-making through supporting cognition and shifting the patient’s role. We also identified scaffolding structures, an analogy of personalized support, as a precondition for these mechanisms and for the overall feasibility and acceptability of iTandem.

**Conclusions:**

iTandem was generally perceived as a feasible and acceptable tool in the treatment of patients with psychotic disorders. Our findings suggest that nonclinical aspects, such as support structures, are important to the feasibility and acceptability of such digital interventions and patients’ aptness for digitalized treatment in general. Future research should explore related nonclinical aspects further instead of defining potential target groups based on diagnoses and symptom severity alone.

## Introduction

### Background

The clinical integration of technology into health care services has been suggested as a strategy to enhance both treatment efficiency and service user satisfaction [1,2]. Moreover, shared decision-making (SDM) has become a core principle in the design of new person-centered services within mental health care [3,4].

SDM involves mutual involvement and collaboration between the patient and service provider, enabling a shared understanding to explore and decide on therapeutic alternatives throughout the course of treatment, in accordance with patient needs and preferences [5,6]. To achieve a shared understanding of the clinical condition, as well as relevant factors that should be included in the decision-making process, delivering information in a tailored and accessible format is crucial [7,8]. Although the body of available literature is inconclusive, studies indicate that higher levels of patient involvement in mental health care services result in improved treatment outcomes related to both clinical recovery and patient satisfaction [9,10]. Thus, mental health care services are making efforts to develop and distribute publicly available communication tools that promote SDM implementation in routine health care, both in Norway [11,12] and internationally [4].

However, the process is slow, and mental health care services have so far implemented SDM only to a limited extent [13,14]. The research literature indicates that SDM is especially lacking in the treatment of severe conditions, such as psychotic disorders [15,16]. According to a Norwegian study, patients with psychotic disorders report that they are not involved in their treatment as much as they prefer [17]. These findings are mirrored in the international literature [16,18,19]. Nevertheless, a recent study found that receiving information and feeling understood are fundamental care needs of individuals who have experienced a psychotic episode for the first time [20]. Both of these aspects are core elements of SDM and underline the potential clinical value of implementing SDM in this patient population.

There are several obstacles to the implementation of SDM in the treatment of patients with psychotic disorders. Many patients endure cognitive and negative symptoms, which comprise problems such as reduced memory, levels of engagement and interest, and difficulties expressing emotions and needs [21-23]. The presence of such symptoms may impede patients from pursuing a role as active agents who take part in informed dialogues with clinicians. In addition, research has revealed stigmatizing views toward persons with psychotic disorders among mental health service providers [24] and that clinicians expect that patients with psychotic disorders are less able and less willing to engage in a collaborative approach to treatment [25,26]. A possible consequence is reduced effort from the service provider to achieve SDM. Another consequence is that patients perceive the stigma from service providers and feel disempowered [27], which further complicates their opportunities to experience SDM. Furthermore, the services delivered to people with severe mental disorders are complex and resource-intensive activities [28,29]. Patients are typically followed for longer periods of time, and services should be able to adjust the goals and content of the treatment according to changing needs. Without strategies to maintain a focus on common goals, this might be challenged in a protracted course with changing clinicians, departments, and patient contexts [30-33].

The discrepancy between the ideal of SDM-based clinical practices and the current situation in severe mental health care exposes a need to develop novel strategies to enhance patient involvement. Integrating digital tools may offer a flexible and personalized approach to targeting SDM [34]. Highly accessible digital tools, such as mobile apps, offer functionality that can overcome some of the obstacles to SDM. Apps can facilitate the collection of real-time data points, providing rich information about specific aspects of the user’s life, which can be of importance for the process of recovery. In addition, this functionality can reduce the demands on memory and help users convey relevant information during therapy sessions [35,36]. Several app interventions developed for patients with psychotic disorders have been evaluated as feasible and acceptable by users, such as Actissist (University of Manchester), FOCUS (University of Washington Psychiatry & Behavioural Sciences), and PRIME (Personalized Real-time Intervention for Motivation Enhancement; Regents of the University of Minnesota) [37-40]. However, few mobile apps directly target SDM in mental health [34]. The existing SDM apps offer functionality such as mapping clinical decisions and weighing the benefits and disadvantages connected to them [41] or prompting evaluations of therapy sessions and monitoring of health parameters, which can be used to support decision-making [42,43]. Despite the relevant functionality and promising user experiences, adoption and diffusion of such apps remain low in routine clinical settings [44,45]. A user-centered approach that involves service providers and patients from early exploratory stages is necessary to ensure feasibility and clinical benefit, which facilitates diffusion in everyday clinical settings [46].

The idea of the mobile app examined in this study developed from a previous innovation project in which persons with lived experiences stated that they missed alternative ways of collaborating with services and that there is a lack of tools to strengthen the focus on treatment goals during and between treatment sessions [47]. This insight sparked an iterative and person-centered process of concept and software development, involving a thorough mapping of the needs of patients with severe mental illness and their therapists. The outcome was iTandem, a mobile app designed to enhance the patient’s voice in decisions concerning focus areas and treatment goals. Before planning the present exploration of the feasibility and acceptability of iTandem in clinical settings, the app was beta-tested, and its functionality was refined according to feedback from patients and clinicians.

### Study Aim

We aim to explore the feasibility and acceptability of the mobile app iTandem [47] in the treatment of patients with psychotic disorders from the perspectives of both clinicians and patients. In addition, we aim to explore mechanisms that potentially contribute to achieving SDM when using iTandem.

## Methods

### Setting and Recruitment

The study took place at 2 clinical sites at Innlandet Hospital Trust in Norway from December 2022 to May 2023. Innlandet Hospital Trust is part of the public specialist health care services and offers both inpatient and outpatient treatment. The catchment area is geographically large and mainly rural. The hospital trust serves people from a broad range of social strata, and many patients live far away from the specialized services.

One of the sites is a specialized unit for the assessment and treatment of early psychosis. The other site is a psychosis treatment clinic at a district psychiatric center. All the clinicians who participated in the study were invited by the heads of their respective units.

**Figure 1.**
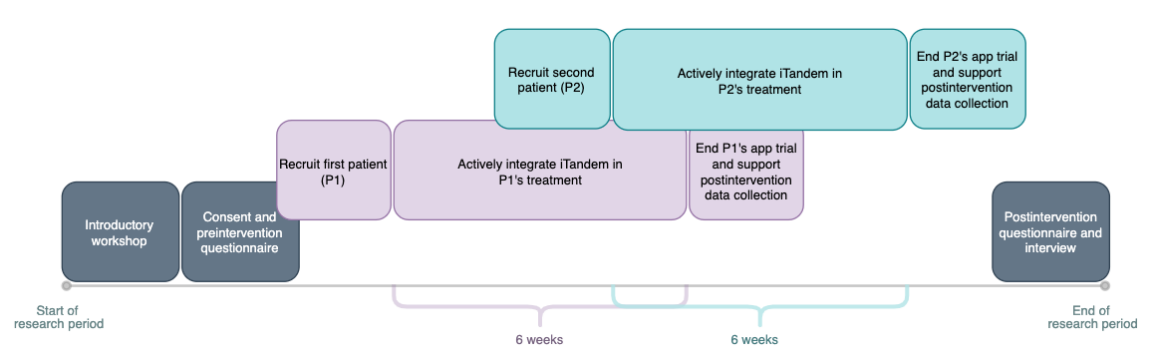
Research activities and project timeline for clinicians participating in the study.

The core project group (consisting of authors MS, SRA, KLR, and GEB) arranged an introductory workshop for clinicians, and the overall aims of the study were explained. The research activities and a timeline of the project are described in Figure 1. Demonstration videos covering the different functionalities of iTandem and providing examples of how to inform and recruit patients were presented. Based on previous user testing, clinicians were advised to guide patients to reduce the selection of modules to 2‐3 to avoid overload. Written instructions for recruitment and how to access a visual report of app data were handed out, including references to the project website [48]. Guided role-play exercises were offered to the clinicians. However, the role-play training was not carried out because the clinicians did not perceive it to be necessary. Clinicians were encouraged to contact the project coordinators (MS and GEB) when they needed support. GEB was a local coordinator.

Initially, 10 clinicians agreed to take part in the study. They participated in an introductory workshop and signed an informed written consent form. Eight completed the trial, while the remaining 2 clinicians left the study due to lack of engagement in patient recruitment and personal reasons, respectively.

Patients were invited by their clinicians to participate in the study during routine consultations. The inclusion criteria for patients were as follows: currently undergoing assessment or treatment for a psychotic disorder, age 18 years and older, having access to a smartphone, being able to download the iTandem app, and being willing and able to consent. The first inclusion criterion, concerning the presence of a psychotic disorder, was based on clinician ratings. Experienced clinicians working at the clinical sites of this study assessed and diagnosed patients using the *International Classification of Diseases, 10th Revision* diagnostic criteria for schizophrenia, schizotypal, and delusional disorders (F20-F29). The 2 clinical sites offer services exclusively to patients diagnosed with a psychotic disorder and patients undergoing assessment based on indications of a psychotic disorder. Therefore, all patients included in the study met this criterion.

The convenience sample of patients first comprised 14 patients from both outpatient and inpatient units. During the trial, 5 left the study. Two patients did not complete the trial because their therapists were absent from the clinic for much of the trial period due to vacation or sick leave. One patient agreed to participate but never began using the app actively. One patient was discharged and left the study, while another patient did not attend the sessions within the trial period.

Six patients and all 8 clinicians agreed to participate in interviews. A total of 2 patients and 2 clinicians were interviewed in person, 2 patients were interviewed over the phone, and the remaining participants were interviewed through a secure video platform. The interviews were performed by MS and KLR.

### Intervention

The iTandem mobile app [47] is developed by the Early Intervention in Psychosis Advisory Unit for Southeast Norway and the app development team at the Information Technology Department at the University of Oslo. iTandem is designed as a supplement to typical treatment. The purpose of integrating the app in treatment is to enhance therapeutic communication and accentuate the needs and preferences of patients in treatment for severe mental disorders or addiction. Since iTandem is designed for personalized and need-based use, there are no generic recommendations concerning the ideal combination of modules or how long the app should be an integrated part of treatment. However, the trial period of this study was set to 6 weeks.

**Figure 2.**
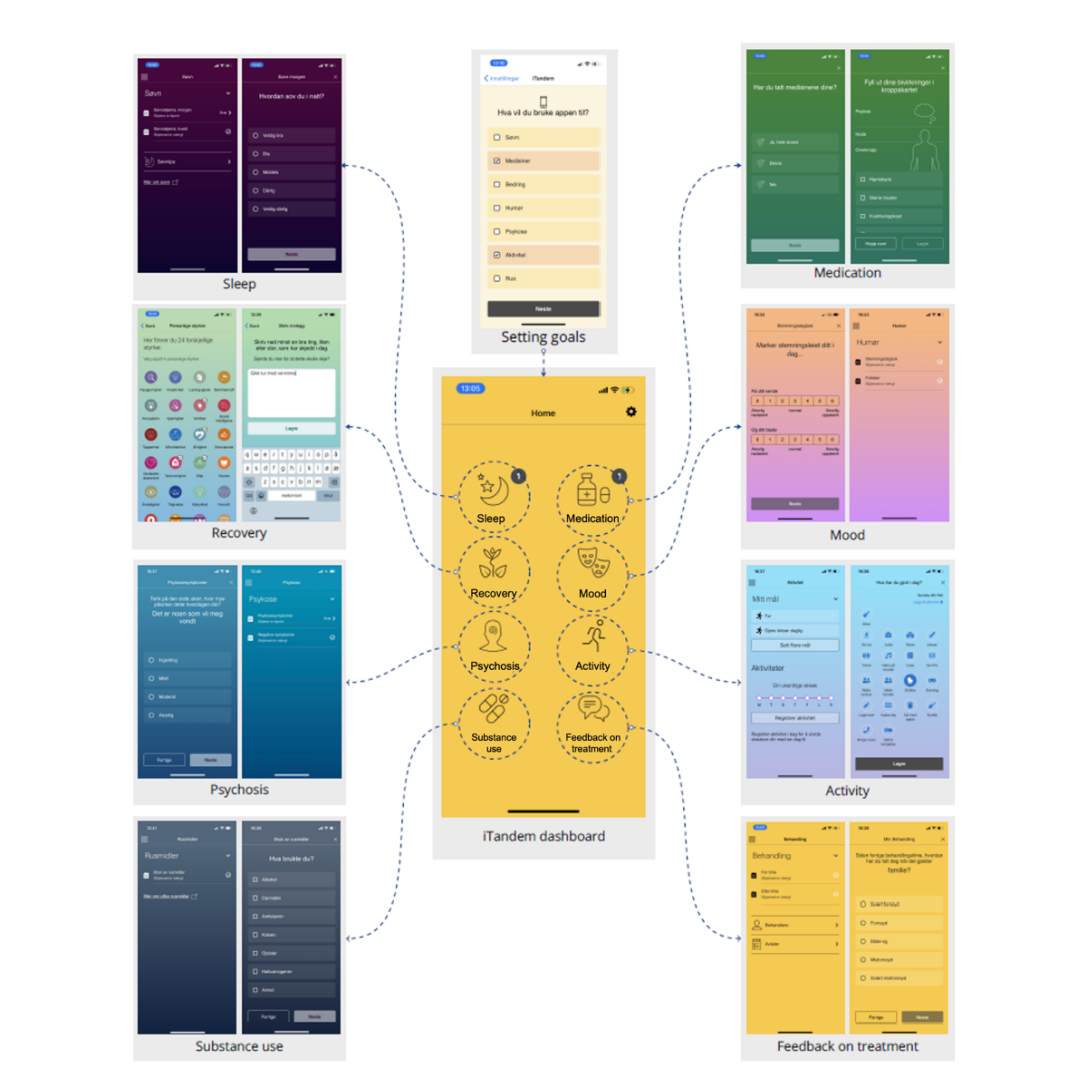
The dashboard and modules of the iTandem app.

iTandem is administrated by patients, while their clinicians have access to a digital report that summarizes the app data. iTandem contains 8 optional modules assessing sleep, medication, recovery, mood, psychosis, activity, use of substances, and feedback concerning treatment (Figure 2). The contents of the different modules are described in detail elsewhere [47]. The app prompts patients to register data regularly. The time intervals between notifications depend on which modules the user has activated. For instance, the sleep module entails a sleep diary that requires data registrations in the morning and in the evening. When this module is activated, the user receives notifications twice a day. In contrast, the psychosis module sends out weekly prompts, as the symptoms tracked with this module change more slowly and over a longer period of time.

In this study, patients consented to participate in the 6-week trial via a web-based form with guidance from their therapists. Thereafter, patients downloaded the iTandem app on their personal smartphones and discussed with their therapists which of the modules would be most meaningful to their condition and current life situation. This conversation represents the beginning of an SDM-based approach, directing the focus of ongoing treatment in accordance with patient needs and preferences. Patients and clinicians were encouraged to change the selection of modules during the six-week trial period if they experienced that a treatment goal was achieved or perceived other modules as more useful.

**Figure 3.**
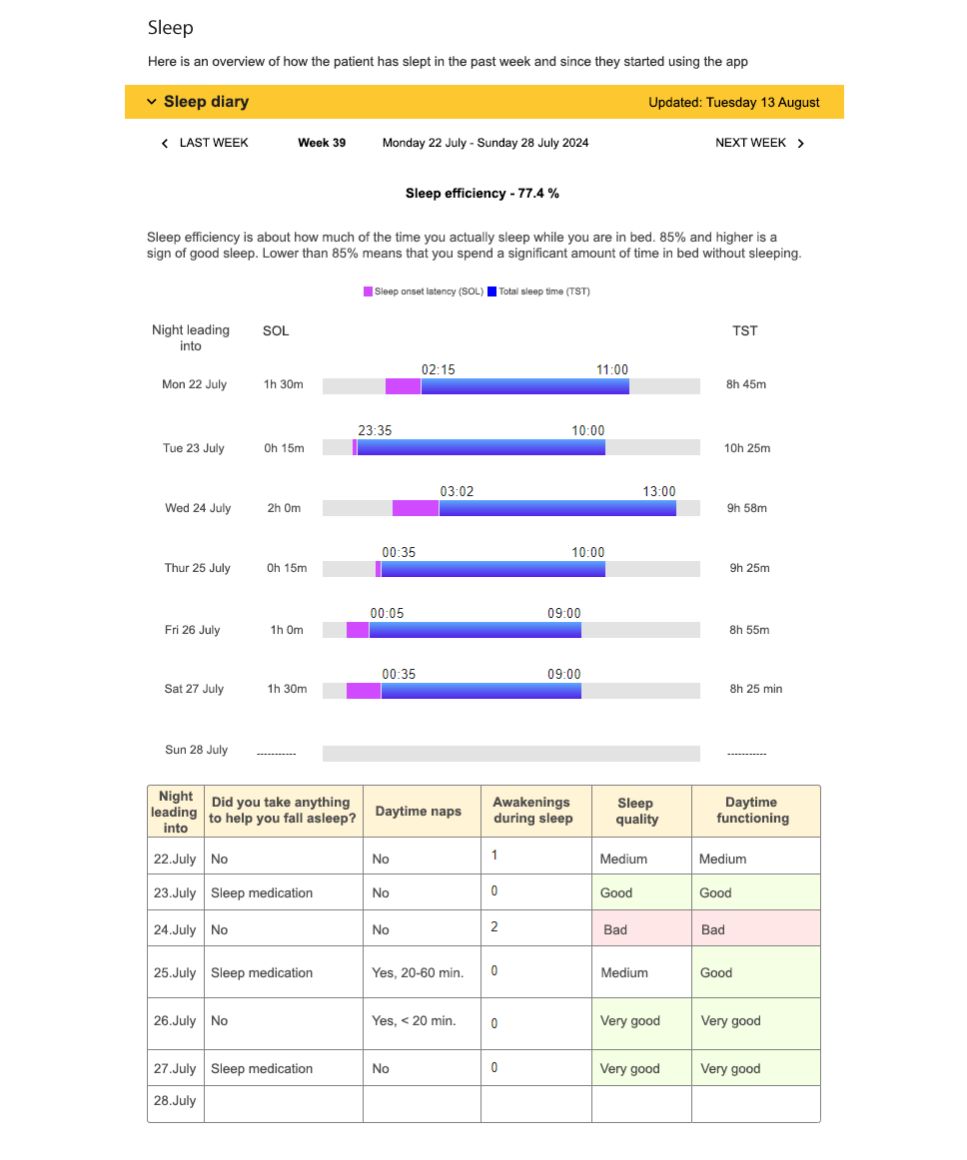
The sleep module is visualized in the report. The content has been translated into English.

The data registered by patients during the trial period were transferred directly to Tjenester for Sensitive Data*,* a service for storing and processing sensitive data at the University of Oslo. These data comprised the basis for an automatically generated digital report with visual representations of the active modules. Clinicians accessed the report through a secure web portal. Figure 3 demonstrates how the sleep module is represented in the report, summarizing a week of data registration.

**Figure 4.**
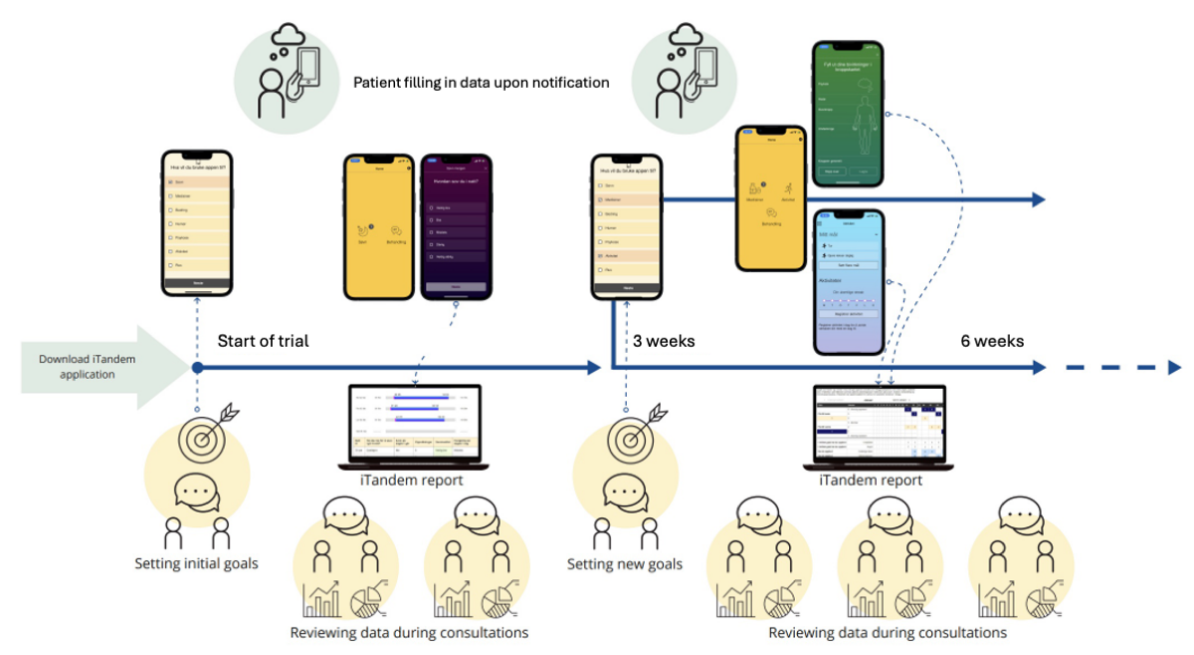
Flexible integration of iTandem throughout the trial period.

In the introductory package, the clinicians were instructed to share and discuss the report with their patients during the treatment sessions of the trial period. The project group presented various potential areas of application, such as monitoring fluctuations of symptoms and side effects from medication, identifying connections between different aspects of the patient’s condition and daily life, and guiding ongoing clinical interventions in accordance with reliable patient data over time. Figure 4 demonstrates the process of integrating and actively using the app and report during the trial.

### Study Design

A mixed-methods research design placed within a pragmatic paradigm [49] was applied to explore iTandem’s feasibility and acceptability and the mechanisms potentially involved in achieving SDM. The study places a heavier emphasis on qualitative methods, prioritizing active researcher interpretation and broad exploration of individual experiences from a small sample of participants. The quantitative methods of the study comprise some instruments providing reliable data that can easily be compared to benchmark values. Other measures that are typically operationalized in a largely quantitative manner within similar research contexts involve more qualitative assessments in this study.

The research design applied here largely resembles a convergent design with a side-by-side comparison approach [49], since the data collection and analyses are mainly conducted separately before integrating the findings in the discussion, where the qualitative elements elaborate and nuance the quantitative results. However, the quantitative data are also used to guide the qualitative methods earlier in the analytic process.

### Quantitative Data and Methods

The quantitative data include responses to the web-based preintervention and postintervention questionnaires and app usage data. All 9 participants and 8 clinicians completed the preintervention and postintervention assessments, except for one clinician who did not fill out the postintervention questionnaire. Descriptive analyses were conducted using SPSS Statistics 29 to depict the groups of participants, identify tendencies in the data material, and create a foundation for the qualitative exploration of feasibility and acceptability. The following quantitative measures were included in the study:

#### Demographics and Participant Background Information

In preintervention questionnaires, patients reported gender, age, treatment level (inpatient or outpatient), and duration of treatment. Clinicians reported gender, age, which service they worked in (inpatient, outpatient, or both), and patient diagnoses.

#### Usability

User-friendliness (ie, technical feasibility) was measured using the System Usability Scale (SUS) [50]. Separate items constructed by the project group mapped occurrences of technical errors.

#### Acceptability

Acceptability was assessed with postintervention closed-ended questionnaire items. Both groups were asked whether they experienced the intervention as clinically valuable and to what extent the digital tool had been used during the trial period. In addition, patients reported whether the intervention was experienced as comfortable and safe and which modules they had chosen. App usage data supplemented the patient acceptability measure with indications of whether the participants were engaging actively in iTandem or not. These data were obtained manually by observing and documenting the number of data points registered by each of the participants in the different modules, providing insight into broad tendencies of app activity across the sample.

### Qualitative Data and Methods

The main data source in the current study was the qualitative data material. It comprised both text responses to open-ended items in the preintervention and postintervention questionnaires and individual semistructured postintervention interviews. All 8 clinicians and 6 of the patients participated in interviews. The number of words in the 14 interview transcripts ranged from 2304 to 6574, with a median of 3603 words.

A reflexive thematic analysis was conducted using NVivo 14 (Lumivero). The purpose was to gain a deeper understanding of the feasibility and acceptability measures and to explore these data further, identifying mechanisms that potentially contribute to the intended effect of the iTandem intervention:

#### Feasibility

Feasibility was explored in postintervention interviews. Both groups were asked similar questions about how the iTandem intervention fit into the current clinical setting, what factors may impact a successful implementation, and their thoughts on the future use of apps in long-term follow-up. All participants were asked about potential areas of improvement related to the tool and its implementation. The patients’ postintervention questionnaires included text response items mapping experiences with the specific modules they had used, which were used as an indication of whether the different modules had been applied as intended.

#### Acceptability

The postintervention interviews provided oral accounts of the participants’ enthusiasm, satisfaction, potential negative events, and experience value from taking part in the trial. The patients’ open-ended questionnaire items prompted the patients to share perceived health effects, as well as positive and negative aspects of the intervention.

The thematic analysis of this study is largely based on Braun and Clarke’s reflexive thematic analysis, applying essentialist and experiential theoretical assumptions [51]. The data collection and analysis of the study were guided by the overall aims of the research project, broadly exploring the feasibility and acceptability of iTandem, as well as mechanisms related to SDM. However, the thematic analysis applied here is essentially inductive rather than theory-driven.

MS conducted the analysis. KLR and SRA contributed in stages 1, 4, and 5. The authors involved in the analysis had several explicit discussions concerning aspects that may have had an impact on the methodological choices and results of the study, such as roles, backgrounds, and motivations in the project. KLR and SRA were central actors in the development of the app. KLR, SRA, and MS hold relatively optimistic views on the potential value of digitalization and SDM in health care. These contextual and personal factors impact all stages and decisions of the research project. Furthermore, the participants’ accounts represent subjective interpretations of experiences within specific environments, which are accepted and included in the analysis. Thus, principles of reflexiveness, attention to context, and an experiential approach to data are integral in this application of thematic analysis [52]. The 6 steps of thematic analysis were conducted in a dynamic manner, moving back and forth between the stages described below, especially from the third to the sixth phases:

##### Data Familiarization

MS conducted verbatim transcriptions of the 14 interviews. MS, KLR, and SRA read and reread the interviews and noted their first thoughts about each interview and the dataset as a whole. The group shared ideas and reflected on the content of the data material. This workshop, as well as the other collaborative activities described in later phases, is not a standard element in Braun and Clarke’s reflexive approach. The purpose of the discussions was not to get closer to objectiveness, as this is not considered an ideal in the reflexive form of thematic analysis [51]. Instead, this was done to spark creativity and reflexiveness in the analytic process. The transcripts were imported to NVivo. Electronic memos based on the initial thoughts about the data material were created and connected to each interview.

##### Coding

MS coded the dataset systematically in NVivo. The coding was conducted in accordance with the guidelines for reflexive thematic analysis, where one subjective coder is sufficient to achieve high-quality output, while aiming for interrater reliability using a second rater would represent a deviation from this branch of thematic analysis [51]. No coding frameworks were applied, and no parts of the data material were excluded from the coding process. Although ideas concerning the research questions emerged in these early phases, MS made efforts to direct the same level of attention to all parts of the texts in all interview transcripts to maintain an inductive approach. However, the process can be said to have been influenced by theory to some extent, as the authors had hypotheses related to the research questions and had experience with the same kind of data material from a previous beta test. Furthermore, the interviews were semistructured, which led the accounts in a specific direction, although question formulations were open, and participants were encouraged to communicate their thoughts without restrictions. This phase resulted in a total of 340 semantic codes.

##### Generating Initial Themes

MS combined different codes into groups that represented units of similar meaning in NVivo. Examples of headings of such code groupings are “Target group” and “Prerequisites of experiencing clinical value.” In this phase, the titles of the candidate theme structures were still descriptive and topic-like rather than conveying shared meaning about essential aspects of the data set.

##### Developing and Reviewing Themes

Maintaining an experiential approach to the data, MS went through the tentative themes and the associated data extracts in NVivo to ensure that the themes reflected what the participants had said. As a result, the themes were merged or reconstructed into new tentative themes. In a new workshop, the groups of codes were presented on paper to KLR and SRA. The authors used this as a starting point to adjust themes and structure them in relation to each other. Some were left out as they were deemed not contributing to the research aims. MS also studied the questionnaire material to evaluate whether the tentative themes reflected the participant perceptions provided outside an interview context. This phase resulted in 3 preliminary themes on core mechanisms potentially involved in facilitating SDM through iTandem.

##### Refining, Defining, and Naming Themes

The themes were reviewed again by MS, KLR, and SRA, given titles, and connected to the most representative quotes of the data material. The group also worked on descriptions of each theme that communicated the intended meaning clearly. As the analytic process was carried out in an iterative manner, MS repeated phases 3, 4, and 5. After another round of consulting with the other authors and reviewing and refining the themes, the final set of themes was completed.

##### Producing the Report

MS initiated the write-up in parallel with previous phases of the analytic procedure, long before themes were finalized. Thus, the data analysis, literature search, and writing processes interacted throughout this research project.

### Service User Involvement

Service user representatives were involved in all stages of this study, from early app development to designing the assessment tools for data collection and writing this paper. Early Intervention in Psychosis Advisory Unit for Southeast Norway, at which several of the article authors are employed, has its own board of service user representatives. In addition, the researchers in this project discussed the research design with a user representative employed at Innlandet Hospital Trust. During the writing process, the authors discussed the interpretation of findings with 2 service user representatives from the Bipolar Association Norway and refined the manuscript according to feedback on themes.

### Ethical Considerations

The patients participating in this study were recruited based on their status as patients in service for severe mental disorders and can be defined as members of a vulnerable population. Involving vulnerable persons in research requires careful ethical considerations, including the protection of the participants' interests and obtaining freely given informed consent. These principles were integral in the design of the study and in the introductory workshop for the clinicians. During the introductory workshop and the further communication with clinicians, they were instructed to clearly explain both benefits and risks related to participation to their patients, as well as stressing that all elements of the study participation were voluntary and that withdrawing would not result in any negative consequences. All participants gave consent to participate in the study through a digital form after receiving information about the project presented verbally and in a text document. The project obtained approval from the data protection officer at Innlandet Hospital Trust (23839981) and Oslo University Hospital, the institution responsible for data processing (22/20426). Importantly, this study primarily investigates digitalized service delivery and user experiences, rather than mental health conditions and treatment outcomes. The Regional Ethics Committee evaluated the study to be outside of their mandate, as the research did not primarily aim to collect and analyze health data or develop new knowledge about health or disease (523626).

## Results

### Quantitative Data

#### Participant Background Information and Demographics

**Figure 5.**
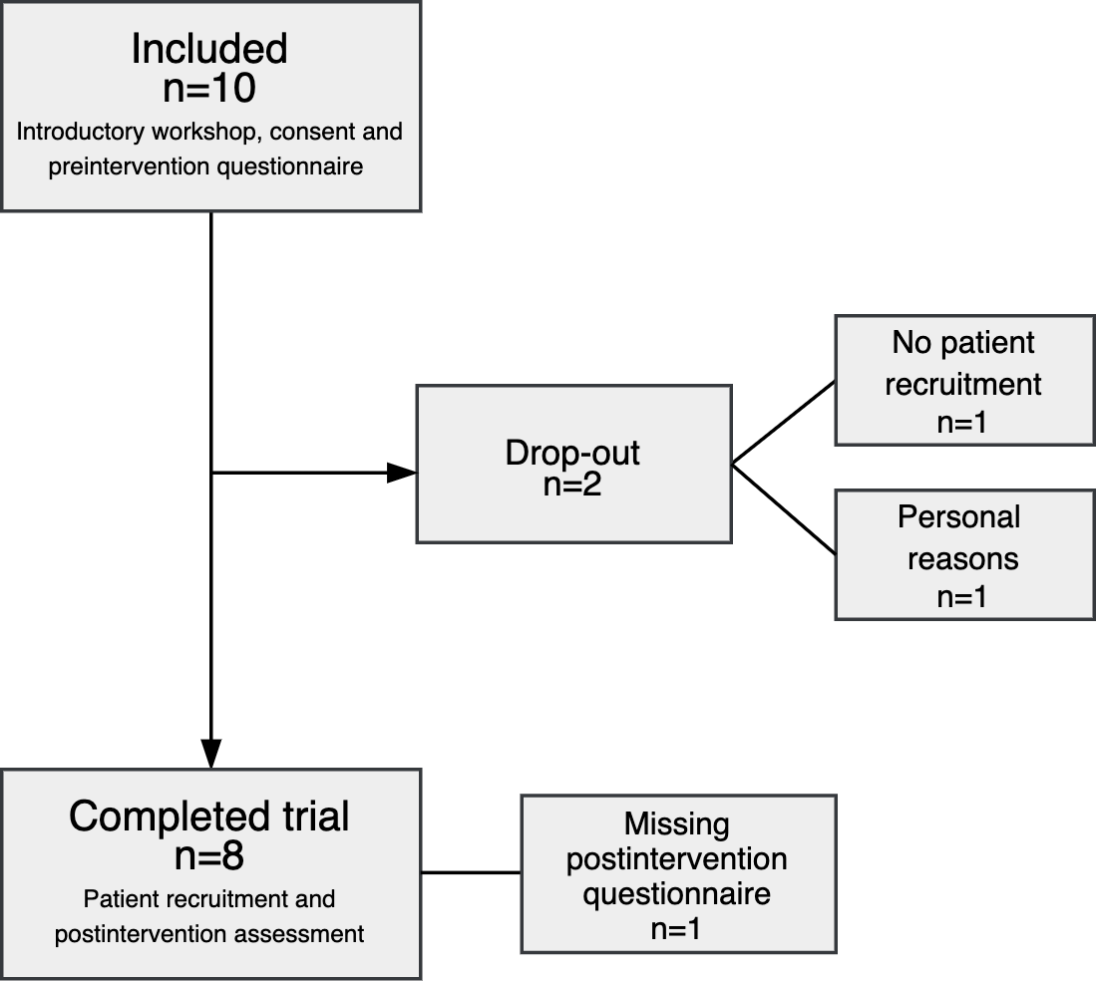
Flowchart of the clinicians recruited to the study.

Eight clinicians completed the study (Figure 5). The clinicians reported the following gender identities: 4 males, 4 females, and 0 other identities. The age range was 27‐63 years old (mean and median 45 y old). A total of 4 clinicians provided outpatient services, 2 inpatient services, while the remaining 2 clinicians worked in both inpatient and outpatient services.

**Figure 6.**
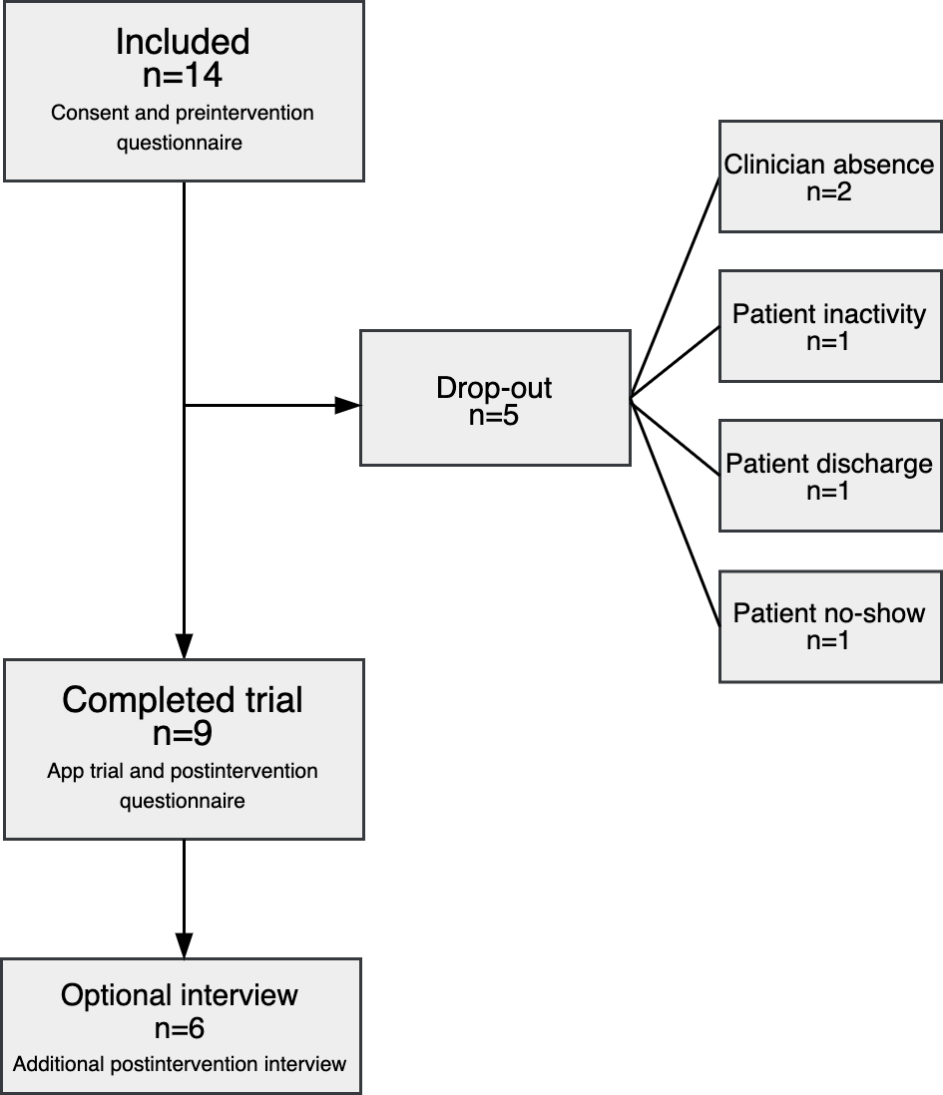
Flowchart of the patients recruited to the study.

Nine patients completed the study (Figure 6). The patients reported the following gender identities: 7 females, 2 males, and 0 other identities. The age range was 18‐43 years old (median 27 y old). Eight out of 9 received outpatient services. The duration of treatment in mental health care services was 0-200 months (median 16 mo). All patients were undergoing assessment or receiving treatment for schizophrenia, schizotypal, or delusional disorders (F20-F29). Two were diagnosed with additional stress reaction disorders and/or a developmental disorder.

#### Usability

iTandem was rated as above average in terms of user-friendliness, with SUS mean scores of 75.0 by patients and 82.1 by clinicians, out of 100.

Additional items covering experiences of technical errors revealed occasions where clinicians were denied access to the report or where the report’s representation of one of the app modules contained errors.

#### Acceptability

The levels of engagement varied across the sample. Most patients engaged with 3 or more modules (Table 1). The introductory package to clinicians recommended limiting the number of modules to one or two, but some patients chose more. Patients displayed a preference for modules related to general health and well-being over symptom-oriented modules. Activity and Sleep were the most popular modules, selected by 8 of the 9 patients.

**Table 1.** Joint display of patients’ questionnaire responses.

Participant number	Selected modules	Report brought up in sessions	Experience of using report in sessions	Experience of sharing personal data	Perceived health effect
103	Sleep, mood, activity	Once	Useful	Ok	Positive: “I became more aware of my feelings and mood … I realized how much I accomplished to do each day. I used the app less after a while because I managed to maintain a good structure in my everyday life. But I liked it a lot in the beginning.”
105	Sleep, mood	Several times	Useful	Ok	Positive: “Provides me with an overview.”
107	Sleep, recovery, activity	Once	Ok	Un-comfortable	Negative: “I became a bit more paranoid and felt watched.”
108	Sleep, recovery, mood, activity, medication, psychosis	Several times	Useful	Safe	Positive: “Maybe a bit, nice to have a routine with the app.”
109	Recovery, activity	Every time	Useful	Ok	Positive: “More aware of activities, that it doesn’t have to be that many every day, but that I still manage some things.”
112	Sleep, mood, activity	No	No experience	Ok	Positive: “It was nice to get an overview of sleep and feelings, and for instance I became more aware that I often go to bed too late in the night.”
113	Sleep, mood, activity, psychosis	Several times	Useful	Safe	Neutral: “No.”
114	Sleep, recovery, mood, activity, medication, psychosis	Once	Ok	Safe	Neutral: “I don’t know.”
115	Sleep, mood, activity	Several times	Ok	Ok	Neutral: “It has not made any big differences to my mental health. It has been the same regardless (…)”

Responses to questionnaire items targeting experienced value from the iTandem intervention revealed distinct differences between patients (Table 1). Five out of 9 patients reported that reviewing the report in sessions felt useful. The same number of patients reported that they experienced positive health effects from using iTandem. Three patients reported neutral experiences with both items. One incident was identified as an adverse event related to the iTandem intervention. One patient participating in the study (#107) experienced increased levels of paranoid thoughts and a feeling of being watched through the digital tool. This patient also reported that sharing data through the report system was experienced as uncomfortable. The patient discussed the problems with their clinician and stopped using iTandem.

Questionnaire data showed that some clinicians were not able to maintain an active focus reviewing the report regularly in treatment sessions (Table 1). Their evaluations of clinical value from the intervention varied. The variation in responses seemed to correspond with the levels of app activity and experienced value from the perspectives of their respective patients.

### Qualitative Data

#### Feasibility and Acceptability

**Figure 7.**
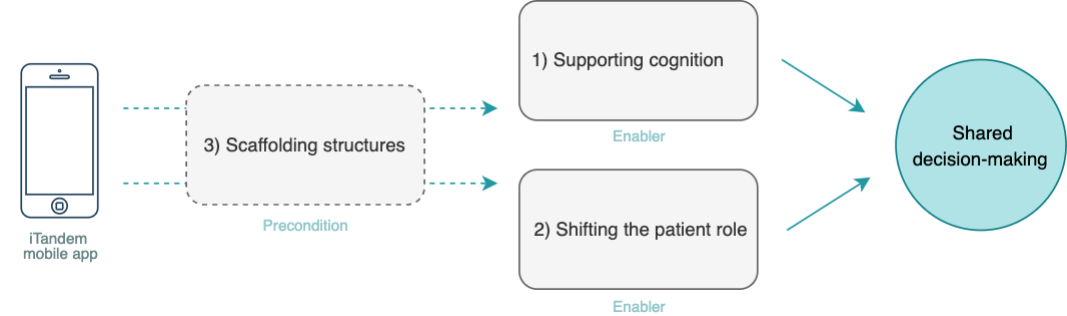
Themes generated through qualitative analysis and their suggested role in promoting SDM using the iTandem app.

Through the reflexive thematic analysis, the following themes, which represent potential enablers for SDM in the iTandem intervention, were generated: Supporting cognition and Shifting the patient role. The third theme, Scaffolding structures*,* was constructed as a precondition for these processes (Figure 7) and suggested as a precondition for the overall feasibility and acceptability of iTandem.

##### Theme 1: Supporting Cognition

The first theme elaborates on how the iTandem intervention served as an aid for cognitive tasks within the treatment context for both patients and clinicians. Participants recognized bias and limitations regarding their own memory, their capacity to communicate inner states, and their ability to follow a structure in both treatment sessions and over a course of treatment. The data registration made by patients in iTandem, together with the clinicians’ use of the report, were viewed as compensatory activities. In addition, several participants expressed that the intervention supplied the treatment with new clinically relevant information and that they had the experience of getting an overview of aspects related to their condition and general well-being.

Graphic representations of the patient data were automatically generated in the report, offering a more accessible format for summarizing and communicating complex information. Thus, iTandem helped translate data about the patient into something easily comprehensible and provided the therapy with more concrete reference points. Participants valued that the visual report provided an overview of parameters related to health and general well-being:

*When patients register their sleep, you access a graph where the information from sleep over time is a lot more comprehensible. It makes it more tangible*.

[Clinician #209]

*It is nice to get to see the patterns, get it documented instead of writing it down or trying to remember it. That does not work*.

[Patient #108]

Some participants talked about the value of iTandem as a tool for clinicians to maintain structure and focus on the patient’s chosen therapeutic project over time. iTandem was also perceived as an aid to promote a more nuanced presentation of the lives of patients. Moreover, iTandem facilitated the search for positive elements in patients’ everyday lives:

*I used iTandem as a reminder for myself to maintain structure and keep track from one session to the next. I think it worked well for remembering to bring up topics. (…)*

*Taking in the nuances in everyday life can be a problem for many patients. One event might be experienced as bad, and the patient focuses on this event only. And then, [with iTandem], it might become evident that there had been better moments during the past week*.

[Clinician #207]

##### Theme 2: Shifting the Patient Role

The second theme covers how the iTandem intervention had the potential to cause a push toward a more active and empowered patient role in therapy. The intervention entailed a direct opportunity for patients to take part in decisions regarding their treatment focus. A patient account reveals that this was experienced as unfamiliar, but the alteration of the patient role resulted in a feeling of involvement and empowerment. Furthermore, when maintaining activity in the selected focus areas over time, the patient experienced increased commitment and agency:

*I felt that I was very involved in deciding what we would focus on. Although it was difficult to express myself firmly, it helped me to think “Okay, this is what I want to focus on, this is what I want.” (…) I liked using the app. In a way, I felt more—I don’t know the exact word, but—I felt that I had to actively use it to get better. Well, there was no pressure, but I felt that it was my responsibility too*.

[Patient #109]

With iTandem, the course of treatment was supplemented with an alternative path for patients to engage in their treatment. Several patients exerted strong digital skills and explored the tool independently. They quickly became comfortable in navigating iTandem, sometimes to a greater degree than the clinicians. Thus, iTandem also represented an area of mastery and a means to show competence and reduce the experience of hierarchies that characterize traditional health care contexts. Furthermore, although most participants across the sample underlined the need for patients and clinicians to know each other well before introducing digital tools in treatment, some talked about technology as a means to relate to and disclose important issues in therapy:

*They grasp it immediately. They are used to apps. I had briefly mentioned the name of the app before I was going to introduce it in the session, and the patient had already registered and started exploring the app*.

[Clinician #211]

*I think [disclosing feelings through an app] is easier. But it depends on who the person is. If it is a friend, I can speak freely. But I don’t feel like I always can do that with a therapist*.

[Patient #112]

Perceiving digital communication as an equivalent or superior alternative to the traditional therapeutic conversation might break with assumptions regarding what high-quality and safe therapeutic communication is for patients in mental health care. Thus, it demonstrates a change in the views of different patient roles and underlines the importance of mapping needs, preferences, and personal traits to create beneficial conditions for secure therapeutic relationships and hearing patient voices.

##### Theme 3. Scaffolding Structures

The third theme refers to support structures that acted as prerequisites for the 2 processes described above and for the participants’ evaluation of iTandem as feasible and acceptable. All the participants talked about social and/or technological structures that had significantly impacted their experiences with iTandem throughout the trial. Examples of scaffolding included the digital infrastructure enabling the operation of iTandem, encouragement to maintain app activity during the trial, and pragmatic strategies to compensate for reduced levels of functioning caused by invasive symptoms.

For some, scaffolding was necessary from the initial use of iTandem. One patient endured paranoid thoughts directed toward digital devices, which made it impossible to use a personal smartphone. To solve this, a relative lent their phone to the patient. The patient expressed positive experiences with the intervention, even though the intensity of paranoid thoughts persisted throughout the trial. Other patients struggled with understanding text and symbols in iTandem and needed guidance from close ones to interpret the content:

*I am positive [toward using the app]. I have not installed it on my phone, I have used [a relative’s] phone. I might have to disclose that first. I have a problem with apps on my own phone*.

[Patient #108]

*I needed to ask my partner at times because I have difficulties understanding the meaning of the tasks*.

[Patient #114]

Moreover, the act of clearly conveying the purpose of the intervention was a central scaffolding prerequisite for both groups. When there was a lack of communication about purpose and use, patients did not necessarily perceive the intervention as helpful, and the clinician was not using the tool to focus the treatment.

Scaffolding structures, such as a safe therapeutic alliance and an attentive clinician, seemed central to the process of enabling a shift in the patient role. Work within the recovery app module provided an example of how scaffolding acted as a precondition for this relational dynamic. The recovery module prompted patients to register positive everyday happenings and personal strengths. These activities appeared to represent a highly relevant focus area in the treatment of several patients and incited encouraging experiences for some. However, without scaffolding, such as the presence of reassurance and guidance from a clinician, the app intervention was not feasible and did not result in experiences of SDM-related processes. The differences across the sample are exemplified by the following quotes:

*I would say that [the recovery module] was the most positive aspect. I got a new perspective on which strengths I actually possess. Especially for someone who doesn’t think they have any…I actually have some*.

[Patient #109]

*With “good experiences” and “personal strengths,” it’s all about positive things concerning yourself, you know. You are meant to come up with positive things about yourself, and that is not something I do.s So that part is difficult for me*.

[Patient #115]

## Discussion

### Principal Findings

This study aimed to explore iTandem’s feasibility and acceptability and the mechanisms potentially contributing to SDM in treatment for psychotic disorders based on a 6-week app trial and data from questionnaires and interviews. The findings reveal largely positive evaluations of user-friendliness and acceptability. SUS scores from both patients and clinicians place iTandem’s usability higher than the benchmark mean score of digital health apps [53]. However, there were substantial differences in how much the app was used and in perceived clinical value, including its contribution to SDM. These variations were explored further through the qualitative analysis. The qualitative findings suggest that the app has the potential to initiate cognitive and relational mechanisms that are central to enabling SDM in therapy. These processes, as well as evaluations of iTandem as feasible and acceptable, often relied on personalized support over time or “scaffolding.”

Our results suggest that a digital tool like iTandem can offer an alternative channel to communicate inner states, as well as a platform where the information is made accessible for both the patient and clinician. Thus, iTandem facilitates a common recognition of clinically relevant issues incited by the patient. The findings fit well with existing literature underlining how cognitively demanding SDM processes can be for patients [54] and highlighting the importance of building a shared understanding to succeed in implementing SDM [7]. Importantly, the provision of information alone is insufficient as a means of driving SDM. In the absence of cognitive aids that help patients apply complex information to their own situation, information does not necessarily contribute to moving the patient toward experiencing more empowerment [55]. Existing literature has pointed out the value of multidimensional information tools in facilitating both the involvement and apprehension of health information in severe mental health contexts [56]. In accordance with this literature, the participants in our trial expressed appreciation for the tables, graphs, and other visual cues of the iTandem intervention. Nevertheless, research on digital SDM tools for mental health care [34,57] has little focus on cognitive mechanisms and the formats of information from the perspectives of both clinicians and patients. An enhanced focus on tools that aim to compensate for limited cognitive capacity may be useful in the further development of digital interventions aimed at strengthening SDM. This may be of even greater relevance to patient populations that struggle with cognitive and negative symptoms [21-23] and their clinicians.

The integration of iTandem caused a movement in the patient role toward increased agency and involvement. The change was facilitated by the introduction of a digital patient-administered approach to relating to and actively impacting the direction of therapy. As the iTandem app entails personalization at the operational level, it is simple for users to tailor the use of the app based on individual preferences [26]. Interestingly, the results revealed a tendency toward preferring modules related to general health and well-being over modules dealing with illness-related symptoms or drug use. This tendency was found in both the quantitative and qualitative data material, and although it is not statistically valid, it may represent the relevance of a holistic and resource-focused approach to treatment. This is in line with a desire for more recovery-oriented mental health care services [58], where we move our clinical focus from only monitoring a reduction in severity of symptoms toward more person-centered treatment goals reflecting what the patients find important for their well-being [59,60]. Thus, efforts to strengthen SDM with flexible and person-centered methods harmonize with the recovery movement of mental health care [9,57].

The patient role shift also accentuates the complex relational processes underlying SDM. Previous research has conceptualized SDM as a method of care [5,6]. This perspective underlines that SDM should be approached as an intrinsic value in therapeutic settings, not a practical procedure. Operationalizing SDM by using digital tools such as iTandem can help us overcome some of the barriers we have been facing until now, especially in vulnerable populations. However, patients hold different preferences regarding roles in the therapeutic relationship, the desire for control in health care settings, and the degree to which they would like to involve themselves in decisions about their own condition [13,61].

The participants’ accounts indicated that various social and technological support structures acted as prerequisites for experiencing the 2 processes discussed above and for the overall feasibility and acceptability of iTandem. There exists evidence that speaks for the value of several types of support in severe mental health care, such as the involvement of relatives in treatment [62,63] and social support in more general terms [64-66]. This literature underlines the general relevance of scaffolding for patients with severe mental health conditions, which proved to be a central concept in our findings. In addition, the importance of supportive social dynamics has been recognized in models from implementation science [67-69]. However, the emphasis in the implementation literature is often on more formal and impersonal support structures, such as the availability of resources and organizational backing. The concept of scaffolding structures in this study is more sensitive to subtle individual differences in social and practical needs, rooted in a person-centered approach.

Importantly, there are prerequisites that are more fundamental than the scaffolding structures discussed above. The target population of iTandem is characterized by a lower level of digital competency and financial status than the general population [70,71]. Although previous research has shown high rates of smartphone ownership within the population [72], there still exists a minority that lacks the required financial means and digital skills, for whom interventions like iTandem remain inaccessible. The presence of paranoid thoughts directed toward technology has been identified as another barrier [72]. Although this barrier does not apply to most patients, the one adverse event in our trial demonstrates the relevancy of recognizing this subgroup. In our trial, the therapist reported the adverse event after it was successfully handled in the treatment sessions and the symptom level had been normalized. The clinician also informed the project group that the therapeutic alliance had maintained cooperation despite the emergence of paranoid thoughts directed toward iTandem. Therefore, we perceived the event’s level of seriousness as moderate and the negative reaction as reversible, and no interventions were initiated. Nevertheless, such adverse incidents expose a need for strategies to uncover and handle negative participant reactions. Guidelines addressing this are currently emerging [73], underscoring the importance of improving future practice.

In the digital mental health field, conditions such as depression and anxiety have been the main areas of focus [74,75]. Our study suggests that digital tools also have the potential to compensate for unmet needs associated with patients with psychotic disorders, such as cognitive and negative symptoms and a pronounced lack of participation in clinical decision-making. Thus, mental health care services should also offer such tools to this patient population. Existing research on interventions that share some of iTandem’s functionality supports this notion [34,76]. For instance, the computer-based decision-making tool TReatment E-AssisT (TREAT; RoQua Inc.) improved clinical discussions by uncovering more clinically relevant topics that aligned with the needs of patients with psychotic disorders [77]. However, the tool did not significantly affect the levels of SDM [78]. This optimistic but inconclusive evidence of digital tools and SDM [34,76] encourages further research on the mechanisms involved. Our study also invites further research on individual differences that are not related to psychopathology, such as the preferred degree of control and decisional impact, tech-savviness, and the digital habits of patients. In addition, we recommend exploring clinicians’ perspectives on SDM and digitalization. Finally, research investigating the feasibility of these concepts at service, economic, and societal levels is needed. We will address some of these knowledge gaps in an ongoing study that includes iTandem and uses a modified study design and new outcome measures.

### Limitations

This is a small study, and the limited number of participants makes it impossible to draw firm conclusions. In addition, technical problems with the report that occurred during the study represent a limitation, as there were cases where interruptions obstructed participants from carrying through the intervention as planned. Another limitation is the possible weaknesses in the introductory workshop. We did not insist on doing role-play to make clinicians more familiar with how it should be introduced when they were reluctant. We believe this could have helped them convey the essential recommendations regarding the need to limit the number of app modules employed by patients. There are also ethical concerns related to the recruitment procedure. Since clinicians recruited their own patients, there is a risk that patients felt obligated to participate because of the pre-established relationship. Importantly, during the introductory workshop for clinicians, the project group communicated the importance of fully informed consent and the option to permanently leave the trial for all participants. The clinicians were also instructed to underscore the voluntary nature of participation when recruiting patients to the study. Nevertheless, research staff will be responsible for patient recruitment in the new trial. Moreover, the study is based on the conception that SDM and clinical app implementation are generally wanted in health care services, and a final limitation is that this stance is not challenged. The mixed methods design is, however, a strength of this study. A pragmatic combination of quantitative and qualitative methods provided the study with a comprehensive and nuanced understanding of the participants’ experiences with iTandem and SDM. The qualitative emphasis allowed us to collect rich data, although the sample was small. Participants perceived the threshold to respond to questionnaires as lower than that of being interviewed; thus, we were able to collect information, even from those who were reluctant to speak with us. Another strength is the naturalistic setting and the inclusion of user perspectives of both patients and clinicians during development and testing.

### Conclusions

Patients with psychotic disorders and their clinicians generally perceived iTandem as a feasible and acceptable supplementary tool for treatment. The app has the potential to serve as an aid for cognition and to facilitate a shift of the patient role toward a more active position in clinical settings. The study suggests that the feasibility and acceptability of iTandem depend on some fundamental prerequisites, including appropriate support structures. We recommend not excluding this patient population from accessing digital interventions based on the severity of the diagnoses and invite further research on how nonclinical individual differences impact experiences with digital tools and SDM. We also encourage research on the digitalization of mental health care and SDM that explores service, economic, and societal domains.

## Abbreviations

ICD-10: International Classification of Diseases, 10th Revision
PRIME: Personalized Real-time Intervention for Motivation Enhancement
SDM: shared decision-making
SUS: System Usability Scale
TREAT: TReatment E-AssisT
